# Complete response to third-line treatment with trifluridine/tipiracil (TAS-102) in stage IV colon adenocarcinoma

**DOI:** 10.37349/etat.2023.00136

**Published:** 2023-04-27

**Authors:** Celia Lara-Morga, Magda Palka-Kotlowska, Sara Custodio-Cabello, Vilma Pacheco-Barcia, Luis Cabezón-Gutiérrez

**Affiliations:** 1 Facultad de Medicina, Universidad Francisco Vitoria, 28223 Pozuelo de Alarcón, Madrid, Spain; 2 Medical Oncology, Hospital Universitario de Torrejón, 28850 Torrejón de Ardoz, Madrid, Spain; University of Campania “Luigi Vanvitelli”, Italy

**Keywords:** Complete response, colorectal cancer, trifluridine/tipiracil (TAS-102)

## Abstract

A clinical case of a 61-year-old female diagnosed with stage IV right colon adenocarcinoma (unresectable liver and multiple lymph node metastases at the time of diagnosis), Kirsten rat sarcoma viral oncogene homolog (*KRAS*), neuroblastoma rat sarcoma viral oncogene homolog (*NRAS*) and v-raf murine sarcoma viral oncogene homolog B (*BRAF*) wild-type, proficient mismatch repair (pMMR), in whom a complete response to the third-line of systemic treatment with trifluridine/tipiracil (TAS-102) was obtained. The complete response has been maintained for more than 2 years after its suspension.

## Introduction

Colorectal cancer (CRC) is the third most common cancer in the world [1]. In 2022 in Spain, it is expected to be the most diagnosed tumor in both sexes, after prostate cancer in men and breast cancer in women [2]. The average age of presentation of CRC is 70 years although diagnosis in younger patients has increased in the last years [3].

Among the risk factors for developing CRC are dietary factors such as a high-fat diet and a low intake of vegetables and fruits which can increase the risk of polyps in the colon and/or rectum and inflammatory bowel diseases. Approximately 25% of patients diagnosed with cancer have a family history of CRC, although only a 10% of these tumors have a hereditary component [3].

Among people diagnosed with colon cancer, 20% have metastatic CRC [4], and 40% present with recurrence after previously treated localized disease [5]. Kirsten rat sarcoma viral oncogene homolog (*KRAS*) gene mutations are present in 40% of CRC patients and have been identified as a negative prognostic biomarker [6]. The vast majority of *KRAS* gene mutations occur at codons 12 and 13 of *KRAS*. v-raf murine sarcoma viral oncogene homolog B (*BRAF*) mutations have been described in 15% of and confer a worse prognosis than mutations in the *KRAS* gene for CRC [6]. Fifteen pecent of localized colorectal carcinomas have microsatellite instability which has a predictive value for resistance to adjuvant chemotherapy-based therapy, but nevertheless, these types of tumors have a potential response to immunotherapy [7].

Prognosis and survival depend on the CRC molecular subtype which is associated with the tumor’s natural history and can predict response to systemic therapy regimens. Patients with *KRAS*/neuroblastoma rat sarcoma viral oncogene homolog (*NRAS*)/*BRAF* wild-type metastatic CRC, median survival with treatment is approximately 30 months, with survival rates of 80% at 1 year, 40% at 3 years and 20% at 5 years after the start of first-line chemotherapy [8].

The case presented is of special interest as it describes the complete response—as has been histologically confirmed—to trifluridine/tipiracil (TAS-102) in a patient with stage IV right CRC who did not undergo surgery of either the primary tumor or its metastases. To date, no similar case has been described with TAS-102, and the complete response rate with this drug is 0%.

Complete response rates for unresectable stage IV CRC are anecdotal, especially in the context of third-line treatment. However, the case present is based on a patient in whom a confirmed and maintained complete response to TAS-102 was achieved.

## Case report

### Case pesentation

**Figure 1.**
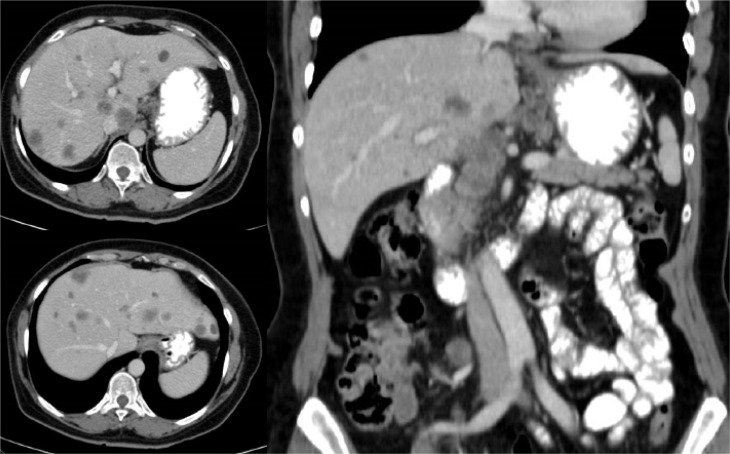
Basal CT scan. Multiple and bilobar hepatic metastases are observed

A 61-year-old Caucasian woman with no medical history of interest was admitted to the internal medicine department of the Hospital Universitario de Torrejón due to pain in the right iliac fossa with no other symptoms. Abdominal ultrasounds showed liver lesions and the computerized tomography (CT) thoracoabdominal-pelvic scan showed an irregular thickening of the cecum wall with locoregional adenopathies in both the hepatic hilum and in the gastroduodenal ligament and multiple hepatic metastases (Figure 1). Blood tests revealed high tumor markers in the carcinoembryonic antigen (16 μg/L), cancer antigen 125 (CA 125; 56 μg/L), CA 15.3 (33 μg/L), and CA 19.9 (> 700 μg/L). Colonoscopy revealed a stenotic ulcerative neoformation of approximately 3 cm in diameter that prevented the passage of the endoscope. Biopsy confirmed an adenocarcinoma of the right colon *KRAS*, *NRAS* and *BRAF* were wild-type.

### Outcome and follow-up

Given the diagnosis of unresectable proficient mismatch repair (pMMR), rat sarcoma (*RAS*), and *BRAF* wild-type stage IV right colon adenocarcinoma (multiple extra-regional abdominal lymph node metastases and bilobar hepatic metastases), the multidisciplinary tumor committee decided to treat with first-line palliative chemotherapy 5-fluorouracilo, leucovorin and irinotecan (FOLFIRI)-cetuximab scheme [cetuximab (Merck Europe B.V, Erbitux, 5 mg/mL, Vial 20 mL) 500 mg/m^2^ on day 1, irinotecan (Irinotecan Accord 500 mg, 20 mg/mL, Vial 25 mL) 180 mg/m^2^ on day 1, folinic acid (Folinato Calcico, 350 mg Polvo Vial) 400 mg/m^2^ on day 1, and fluorouracil (Fluorouracilo Vial, 5 g, 50 mg/mL, 100 mL) 400 mg/m^2^ intravenous bolus on day 1, then 2,400 mg/m^2^ over 46 h continuous infusion on day 1] in an attempt to increase the response rate to stenosing neoplasm.

**Figure 2.**
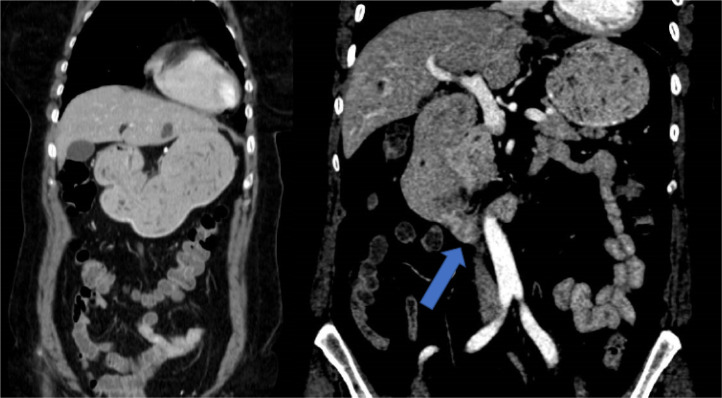
Urgent CT scan showed pseudonodular hypodense image depending on the lateral wall of the second duodenal portion (blue arrow) that causes distension of the gastric chamber

A major partial response was obtained as the initial response, with a complete hepatic response after 6 months of treatment. Nodal progression was also observed after a progression free survival (PFS) of 12 months (appearance of adenopathy conglomerate in the root of the mesentery). The patient presented a grade 2 acneiform rash and grade 2 afebrile neutropenia as maximum toxicity. A second-line palliative chemotherapy 5-fluorouracilo, leucovorin and oxaliplatin (mFOLFOX6)-bevacizumab scheme was then started [intravenous infusion of 5 mg/kg of bevacizumab (Zirabev, 400 mg, 25 mg/mL, Vial 16 mL) during 30 min, followed by an 85 mg/m^2^ intravenous infusion of oxaliplatin (Oxaliplatino Accord, 200 mg, 5 mg/mL, Vial 40 mL) given concurrently with leucovorin at a dose of 200 mg/m^2^ during 120 min, a 400 mg/m^2^ intravenous bolus of fluorouracil, and a 2,400 mg/m^2^ continuous infusion of fluorouracil for 48 h, starting on day 1]. After 6 cycles of mFOLFOX6-bevacizumab, a new CT scan was performed, with radiological stability. From this point on, both carcinoma embryonic antigen (CEA) and CA 19.9 returned to normal levels and remained so for the rest of the patient’s evolution. Administering a total of 14 cycles with oxaliplatin resulting in stabilization of the disease was obtained as the best case scenario. Oxaliplatin treatment was later suspended due to established grade 1 neurotoxicity, with maintenance 5-fluorouracilo/leucovorin-bevacizumab. After a PFS of 20 months, the patient maintained clinical response although a low burden retroperitoneal lymph node progression was observed (left para-aortic and interaortocaval), and her treatment regimen was continued. After 19 maintenance cycles and a total treatment time of 22 months for the second-line of treatment, the patient had an intestinal obstruction and a new increase of the left para-aortic adenopathies was observed (50% increase). CT scan showed distension of the gastric chamber and, more strikingly, of the first and second duodenal portion with abundant content, with a pseudonodular hypodense image depending on the lateral wall of the second duodenal portion, in addition to bilateral pulmonary thromboembolism and a single pulmonary metastasis in the right lower lobe of 9 mm (Figure 2). Treatment was started with enoxaparin, dexamethasone, absolute diet, and serum therapy with surgical resolution of the condition. An upper gastrointestinal endoscopy was performed visualizing duodenal stenosis with normal mucosa and biopsies, suggestive of extrinsic compression.

**Figure 3.**
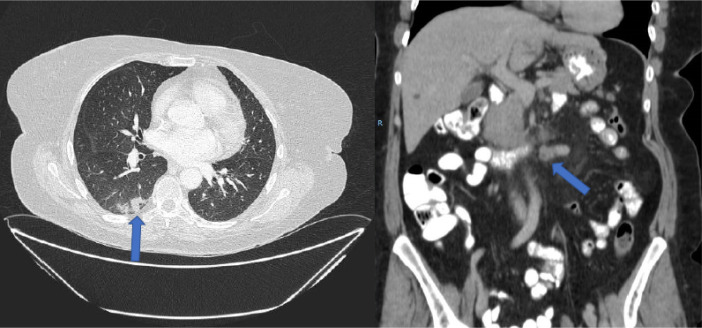
CT prior to the start of treatment with TAS-102. Pulmonary metastasis (blue arrow) and retroperitoneal lymph node involvement are observed. Complete hepatic response is maintained. R: right side of the patient

Given the lymph node progression and duodenal obstruction resolved with medical treatment (with excellent oral tolerance), a third-line treatment with irinotecan plus panitumumab (Amgen Europe B.V; 6 mg/kg of panitumumab and 180 mg/m^2^ of irinotecan, biweekly) was started, but was suspended after the third cycle due to significant toxicity, dizziness with normal blood pressure, grade 2 mucositis, grade 2 rash, grade 2 asthenia and perineal toxicity without radiological progression (Figure 3).

**Figure 4.**
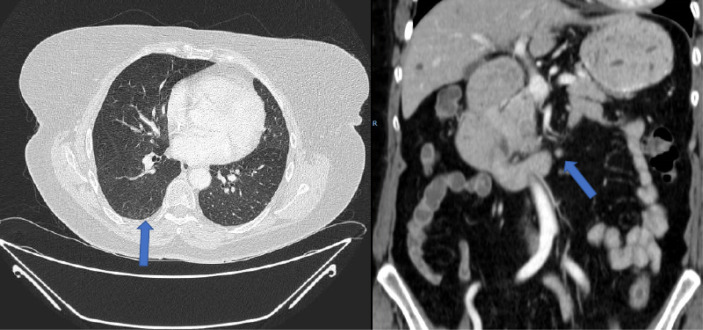
CT after 3 cycles of TAS-102. Complete response of pulmonary metastasis and retroperitoneal lymph node involvement was observed (blue arrows)

A new line of treatment with TAS-102 was started at that time (consisting of 35 mg per square meter administered twice daily, after morning and evening meals, 5 days a week, with 2 days of rest, for 2 weeks, followed by a 14-day rest period). The neutrophil/lymphocyte ratio (NLR) was 2 (< 5). After the third cycle, a complete response of lymph node involvement and pulmonary metastasis response was observed (Figure 4). The complete hepatic response was maintained and after 6 months of treatment, radiological normality was observed clinical complete response.

**Figure 5.**
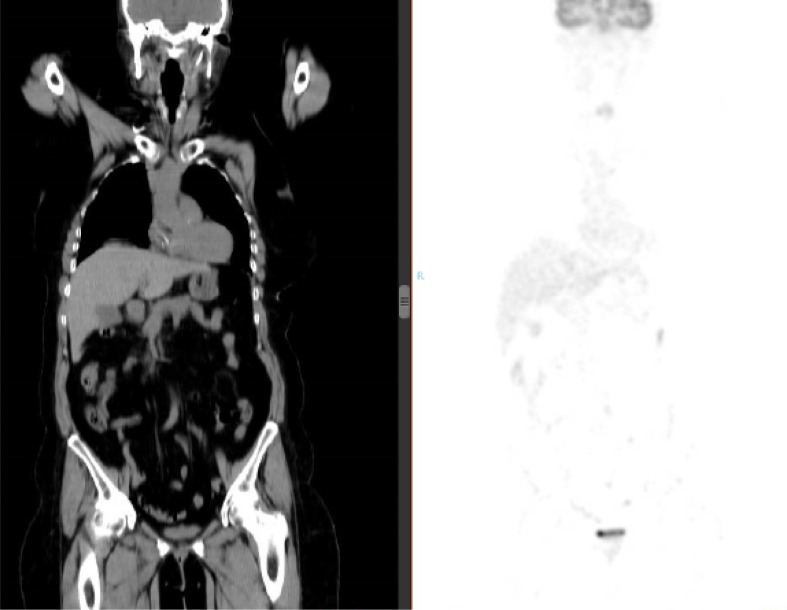
PET/CT showing no pathological uptake of 18-fluoro-deoxy-glucose. Complete biometabolic response is confirmed. Left of the figure is the corresponding CT image. Right of the figure is the image corresponding to the PET, with absence of pathologi-cal uptake

After 29 cycles of treatment with TAS-102, the third-line palliative chemotherapy treatment was suspended after a PFS of more than 2 years. A positron emission tomography-CT (PET-CT) scan (Figure 5) and colonoscopy performed at this time revealed the absence of disease, accompanied by a biochemical response with the tumor marker carcinoembryonic antigen decreased to less than 0.5 μg/L.

A close follow-up of the patient with blood tests and thoracoabdominal CT scan every three months and a colonoscopy one year after discontinuing treatment with TAS-102 is proposed and in all the studies performed to date June-2022, a complete response has been maintained.

## Discussion

Patients with metastatic CRC who experience disease progression despite treatment with FOLFOX, FOLFIRI, and biologic or targeted therapies have refractory disease. There are no approved therapies that have shown to improve survival by 3 or more months for these patients. Management includes palliative care, clinical trial participation, or either of 2 oral Food and Drug Administration (FDA)-approved chemotherapy drugs (regorafenib or TAS-102) [9].

In the RECOURSE trial, TAS-102 improved median overall survival (OS) by 1.8 months compared with placebo [7.1 months *vs.* 5.3 months; hazard ratio (HR) 0.68, 95% confidence interval (CI) 0.58–0.81, *P* < 0.001]. Severe neutropenia (38% with TAS-102 and 0% with placebo) was the most severe important toxicity. The complete response rate is 0%, the partial response rate is 2% and the stabilization rate is 40% [9]. The proportion of patients with “stable disease” is striking (44% for TAS-102 *vs.* 16.3% placebo), which could be postulated to be somehow preventing the accelerated tumor growth that is so characteristic of advanced stages, and which is reflected in the disease control rates shown in the RECOURSE study. However, this result should be considered in the context of at least third-line treatment, in which only a few treatment options with similar results are available [10].

In the CORRECT trial, regorafenib demonstrated a median OS improvement of 1.4 months compared with placebo (6.4 months *vs.* 5.0 months; HR 0.77, 95% CI 0.65–0.94, *P* = 0.005) [11]. The complete response rate is 0%, the partial response rate is 1% and the stabilization rate is 41% [11]. Considering the relative absence of partial and complete responses but the presence of stabilizations, it has been postulated that regorafenib could be prevented in some way of the accelerated tumor growth that is so characteristic in advanced stages [11].

In the FIRE-3 trial, cetuximab plus FOLFIRI demonstrated improved OS compared to bevacizumab plus FOLFIRI (HR 0.76, *P* = 0.012) in *KRAS* wild-type metastatic CRC but no corresponding benefit was found for PFS. The objective of this study was to determine whether cetuximab improves response and survival *vs.* bevacizumab among patients with evaluable response receiving first-line FOLFIRI in *KRAS* wild-type metastatic CRC (mCRC) and the effect of primary tumor side on outcomes [12]. FOLFIRI plus cetuximab resulted in a significantly higher objective response rate (ORR) and longer OS compared with FOLFIRI plus bevacizumab among patients with left-sided tumors. The superior response associated with cetuximab may particularly benefit patients with symptomatic tumors or borderline resectable metastases [12]. The complete response rate is 1–4% for cetuximab plus FOLFIRI in *KRAS* wild-type metastatic colon cancer [12].

The TRIBE 2 study wanted to compare initial FOLFOXIRI plus bevacizumab and reintroduction after progression *vs.* mFOLFOX6 plus bevacizumab followed by FOLFIRIRI plus bevacizumab in the treatment of patients with metastatic CRC. FOLFOXIRI plus bevacizumab demonstrated an ORR of 62% and a complete response rate of 4% [13].

Multiple factors that contribute to tumor spread have been identified, including tumor size, neurovascular or lymphatic invasion, resection margin, sidedness, molecular features, and inflammatory response [14–16]. Indeed, the association between inflammation and carcinogenesis has been widely documented. The activation and proliferation of immune cells associated with a local microenvironment able to sustain specific conditions for tumor growth potentiate the risk of cancer development and progression [17, 18]. Measurement of inflammatory markers has therefore shown promising results in predicting survival in patients with cancer [19]. In recent years, many studies have analyzed survival with respect to the systemic inflammatory response and studied the applicability of predictive immune scores in CRC. Worse survival outcomes have been linked to patients with CRC who have a high NLR compared with a low NLR. That association has been confirmed at various stages of CRC, from early localized disease before surgical resection [20, 21] to more advanced stages [22–24].

Nogueira-Costa et al. [25], conducted a single center, retrospective and descriptive analysis of 102 patients with metastatic CRC treated with chemotherapy and analyzed the potential prognostic value of NLR. Regardless of systemic therapy, approximately 20% of patients underwent metastasectomy. The NLR cut-off was established at 2.35, placing 45 patients in the low-risk group (NLR < 2.35) and 57 in the high-risk group (NLR ≥ 2.35). The Kaplan–Meier analysis showed a median OS of 39.1 months in the low-risk group and 14.4 months in the high-risk group (*P* < 0.001). Multivariate Cox regression on the NLR estimated a HR of 3.08 (*P* = 0.01). The authors confirmed that this biomarker is useful in predicting survival.

This hypothesis is confirmed in the subgroup analysis of the RECOURSE and ROS studies, which evaluate the role of TAS-102 in mCRC [26, 27]. In these cases, the cut-off used is 5. In our patient, the NLR was 2, therefore < 5.

Some cases have been published of patients with metastatic CRC in whom complete response sustained over time has been obtained [28–30], although these are with polychemotherapy with or without targeted therapy schemes and in the first and second-line of treatment.

To summarize, a complete response with TAS-102 in the third-line treatment of refractory metastatic CRC has not been previously described. Molecular subtyping and evaluation of prognostic and predictive factors are critical for the treatment of these patients.

After reviewing the literature, no case of complete response with TAS-102 has been found, hence the exceptionality of this case. Therefore, no possible predictive factors of response have been found that could explain the excellent evolution of the patient.

However, the patient did present some of the predictive factors for a favorable response to systemic treatment to metastatic CRC, which could partially explain the evolution of the patient, such as < 3 metastatic sites, ≥ 18 months from diagnosis of metastatic disease to TAS-102 treatment, RAS and *BRAF* wild-type, left localization of CRC and having NLR < 5.

## Abbreviations

*BRAF*: v-raf murine sarcoma viral oncogene homolog B
CA 125: cancer antigen 125
CRC: colorectal cancer
CT: computerized tomography
FOLFIRI: 5-fluorouracilo, leucovorin and irinotecan
HR: hazard ratio
*KRAS*: Kirsten rat sarcoma viral oncogene homolog
mFOLFOX6: 5-fluorouracilo, leucovorin and oxaliplatin
NLR: neutrophil/lymphocyte ratio
OS: overall survival
PFS: progression free survival

## Declarations

### Copyright

© The Author(s) 2023.
